# Increasing SARS-CoV-2 testing capacity through specimen pooling: An acute care center experience

**DOI:** 10.1371/journal.pone.0267137

**Published:** 2023-06-28

**Authors:** Ana Cabrera, Fatimah Al Mutawah, Mike Kadour, Shannon Schofield, Beverley Conkey, Jeffrey Fuller, Michael Payne, Sameer Elsayed, Johan Delport

**Affiliations:** 1 Pathology and Laboratory Medicine Department, London Health Sciences Centre, London, Ontario, Canada; 2 Pathology and Laboratory Medicine Department, Schulich School of Medicine and Dentistry, Western University, London, Ontario, Canada; 3 Microbiology and Immunology Department, Schulich School of Medicine and Dentistry, Western University, London, Ontario, Canada; 4 Department of Medicine, Schulich School of Medicine and Dentistry, Western University, London, Ontario, Canada; 5 Department of Epidemiology and Biostatistics, Schulich School of Medicine and Dentistry, Western University, London, Ontario, Canada; University of Helsinki: Helsingin Yliopisto, FINLAND

## Abstract

Innovation in laboratory testing algorithms to address seemingly uncontrollable global supply chain shortages in plastics and other consumables during emergencies such as the current COVID-19 pandemic have been urgently needed. We report our experience with specimen pooling on SARS-CoV-2 testing in an acute care hospital microbiology laboratory during a high testing demand period that exceeded available processing capacity. A fully automated four-in-one pooling algorithm was designed and validated. Correlation and agreement were calculated. A custom Microsoft Excel tool was designed for use by the technologists to aid interpretation, verification and result entry. Cost-per-test impact for pooling was measured in reference to the consumable cost and was denoted as the percentage reduction of cost versus the baseline cost-per-test of testing specimens individually. Validation showed a strong correlation between the signals observed when testing specimens individually versus those that were pooled. Average crossing point difference was 1.352 cycles (95% confidence interval of -0.235 and 2.940). Overall agreement observed between individually and pooled tested specimens was 96.8%. Stratified agreement showed an expected decreased performance of pooling for weakly positive specimens dropping below 60% after a crossing point of 35. Post-implementation data showed the consumable cost-savings achieved through this algorithm was 85.5% after 8 months, creating both testing and resource capacity. Pooling is an effective method to be used for SARS-CoV-2 testing during the current pandemic to address resource shortages and provide quick turnaround times for high test volumes without compromising performance.

## Introduction

As of January 24, 2022, there have been more than three hundred and seventy million SARS-CoV-2 infections globally with an associated mortality of approximately two percent [1]. Diagnostic laboratories have faced challenges handling high volumes of specimens submitted daily, unstable reagent and consumable supply chains and laboratory workflow logistics [2–5]. Although global vaccination rates have surpassed eight billion doses, cases continue to spread, coinciding with the emergence of more transmissible SARS-CoV-2 variants [1, 6].

The ability to perform large scale population screening is crucial to ease the lockdown measures [7] necessary to control the different waves of the pandemic. Pooling multiple patient specimens is a well-known technique to conserve resources and has proven to be cost effective. It has been used previously in other types of infectious diseases testing such as screening blood specimens for HIV [8]. This method is more useful in settings where resources are limited but it can be used in any laboratory where sudden surge of testing happens. Pooling was recognized early during this pandemic as a way to increase throughput [9]. The cost savings with pooling varies based on the positivity rate, with about 80% reduction in cost estimated in a population with 1% positivity rate and pooling size of ten specimens in one [10]. In a previous study, it was found that pooling specimens results in 69% increase in testing capacity when the positivity rate is ≤ 10% [11]. Pooling requires no additional equipment but the ability to automate the preparation of the pool is a clear benefit to avoid errors and ease the tracking of specimens. Furthermore, the advantage of screening a larger sample size with improved turnaround time, in addition to savings in reagents and other consumables, showcase the importance of this type of approach now and the future.

The following report describes an in-house developed pooling algorithm that improved workflow logistics, supply and reagent utilization without compromising the analytic performance of the molecular detection method of SARS-CoV-2 used in our laboratory. We comment on the validation strategy, implementation, post-implementation, cost-effectiveness, operational challenges and quality assurance initiatives.

## Material and methods

### Pooling design and validation

The Food and Drug Administration (FDA) recommendations for laboratories included in the “Specimen Pooling” section of the “Molecular Diagnostic Template for Laboratories” guidance document [12] were used for the validation of this algorithm. FDA suggests that five-in-one pooled specimens is a reasonable starting point for validation of pooling for a high-sensitivity test in a population with a positivity rate of approximately 5–6%. We decided on four-in-one pooled specimens for automation simplicity.

### Streaming for pooling

Two specimen streams, individual and pooled, were implemented to optimize the utility of the pooling algorithm to process clinical specimens based on expected positive rate and urgency of the result. The pooled stream was approximately 70% of the volume and included all collections from assessment centers (COVID-19 high-volume community-based ambulatory clinics) and asymptomatic surveillance (outpatients and residents from various institutions). The individual testing stream, approximately 30%, included hospital submissions, symptomatic outbreak investigations, and other high-risk groups. In addition to streaming by source setting, we also refined the streaming by geographic region.

### Pool preparation using high-throughput automation

A Hamilton STARline liquid handler was programmed to prepare a four-specimen pool from primary tubes. The liquid handler recorded the barcode labels of each primary tube and created a report file (the “*pooling reference file*”) with the master list of the primary tubes associated with each pooled specimen. Each pool was prepared with 100 μl of each specimen (total specimen volume of 400 μl of specimen eluted in 60 μl). A Hamilton Vantage 2.0 liquid handler was then used for nucleic acid extraction using Maxwell® HT Viral TNA Kit (Cat No. AX2340, Promega Corporation) and PCR plate preparation. The *pooling reference file* was then loaded into this liquid handler, to produce a final import file for PCR (the *“extraction reference file”*). When tested individually, 200 μl of each specimen is extracted and eluted in 60 μl. It is important to note the overall specimen dilution with this pooling algorithm was 1:2.

### Specimens for validation

To test the extraction efficiency of 400 μL versus 200 μL needed for this algorithm, thirty (30) previously tested positive specimens were selected and extracted in parallel with either 400 μL or 200 μL input. For validation of the pooling algorithm, fifty-five (55) previously tested positive specimens were pooled four-in-one with 321 previously tested negative specimens to produce 94 validation pools. All specimens were selected from a -80°C frozen biobank, tested individually and in pools after thawing.

Specimens included in this pooling study were a variety of upper respiratory tract specimen (nasopharyngeal or midturbinate) swabs transported in various transport media. Transport devices were sorted for pooling and only the same transport media was pooled together. After accessioning in the laboratory, all specimens were heat-inactivated at 56°C for 30 minutes. The main SARS-CoV-2 variants during the time period studied were B.1.1.7 (Alpha) and B.1.351 (Beta). Crossing point (Cp) distribution of positive specimens at the time this study was completed is available in S2 Table. Cp values of all specimens included in this study can be found in S3 and S4 Tables.

### Real-time PCR

A research-use only E-gene / EAV assay (Cat No. 40-0776-96, TIB MOLBIOL) and RNA Virus Master (Cat No. 06754155001, Roche) were used for SARS-CoV-2 screening. The assay is based on the Corman *et al*. E-gene primers [13] and was run on LightCycler 480 II instruments according to manufacturer’s instructions (45 cycles). The *extraction reference file* created during extraction was loaded into the LightCycler at this step to provide the labels associated with each pool. 10 μL of master mix and 10 μl of eluate were used in every reaction. At the end of the PCR run, a results file was exported (the *“PCR results file”*) for automated interpretation downstream. Any amplification curve, regardless of the Cp, was investigated. This assay has been in use in our laboratory since March 2020 and has remained unaffected by the emergence of different SARS-CoV-2 variants. Specimens tested individually using this assay are interpreted as “positive” (detected with a Cp <35), “indeterminate” (Cp 35–39) or “negative” (not detected or detected with a Cp >39).

### Algorithm implementation

A custom Microsoft Excel (2019) tool called BRUT (Batch Results Upload Tool) was designed for use by the technologists to aid interpretation, verification and result entry. The *extraction reference file* and *PCR results file* were loaded into BRUT, populating the tool with specimen identification and PCR curve data including internal control data. BRUT provides automatic application of a wide range of quality rules that are challenging and time-consuming for the technologist including specimen-level control curve checking, run-level quality control checking, PCR cycle threshold summary and specimen ID checking. BRUT proposes the final action for each pool tested and allows the technologist to verify these actions. The primary specimens from the positive pools are returned to the individual testing stream. For the negative pools, BRUT “de-pools” all the data into unique results for each primary tube and allows the technologist to transfer these results directly into the electronic medical record (Cerner Millenium in our institution). The electronic medical record system then automatically sends the results to the ordering physician, the relevant public health unit and the government system which allows the public to directly and immediately access their COVID-19 test result.

### Ongoing quality assurance

Ongoing monitoring of the pooling strategy was implemented by testing a random weekly sampling of specimens without pooling to identify differences in positivity rate between those tested individually and those tested through pooling. This was established to assess false negative rates. To further strengthen our quality assurance initiative, false positive rates were assessed by correlation of positive pools and individual specimen signals. One-week post-implementation data is included in this manuscript, as well as overall performance (September 2020 –May 2021). Pooling was discontinued in late May 2021 as the surge in demand had subsided.

### Statistical analysis

Data plots, including Bland Altman plots, were created on R environment using ggplot2 [14, 15]. Linear regression analysis with y-intercept = 0 was used, slope, correlation, p-value and 95% confidence interval were calculated.

### Ethics statement

This publication is a result of a Quality Improvement initiative and, because of this, no Research Ethics Board approval was sought or obtained.

## Results

**Fig 1:**
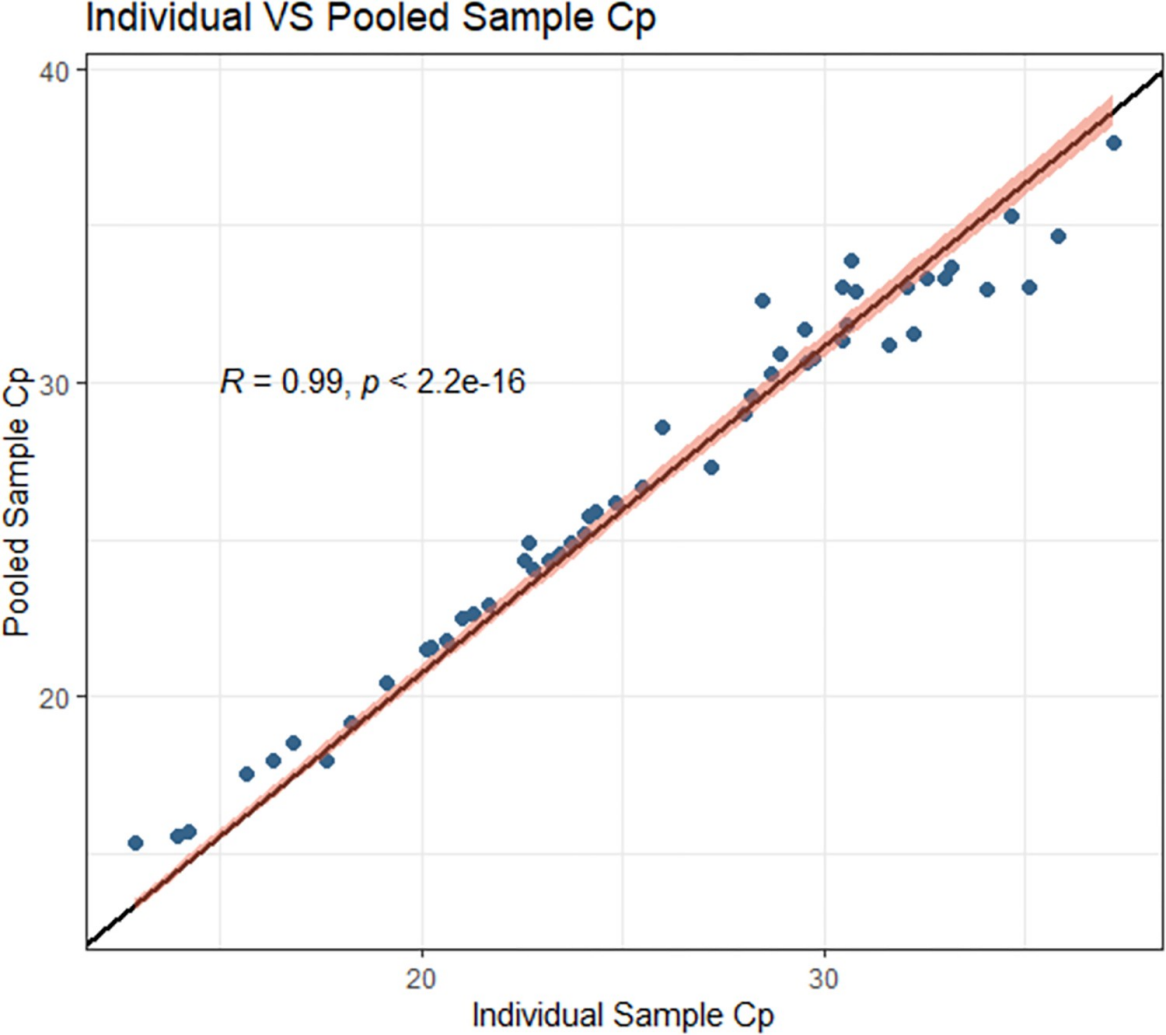
Pre-implementation data plot of the individual specimen Cp and the pooled specimen Cp. Regression line is shown, y-intercept = 0, slope = 1.04745, correlation of 0.9986 and a p-value of < 2.2e-16. 95% confidence interval shown in red.

The extraction efficiency of 400 μL of specimen was satisfactory and comparable to extracting 200 μL alone (manufacturer recommendations), S1 Fig shows a good correlation between results. Using these specimen volumes for extraction, validation of our four-in-one pooling algorithm showed a strong correlation between the signals observed when testing specimens individually (200 μL) and pooled (100 μL of each specimen for a total of 400 μL) (Fig 1). The overall dilution of each specimen in the pool was 1:2.

The average Cp difference was 1.352 cycles with a 95% confidence interval of -0.235 and 2.940. The overall agreement observed between individually and pooled tested specimens was 96.8%. Positive and negative percent agreement is summarized in Table 1.

**Table 1:** Agreement of pooled test result versus expected results.

A) 2x2 contingency table		
	**Expected result**	
**Pooled test result**	**Positive**	**Negative**
**Positive**	52	0
**Negative**	3*	39
B) Performance characteristics		
**Performance characteristic**	**%**	**95% CI**
Positive Percent Agreement PPA	94.5%	85.1–98.1%
Negative Percent Agreement NPA	100.0%	91.0–100.0%
Overall Agreement	96.8%	91.0–98.9%

*False negative specimens had a Cp of 32.13, 36.58 and 39.0

The stratified agreement shows an expected decreased performance of pooling for weakly positive specimens (Cp > 30), dropping below 60% after Cp 35 (Table 2).

**Table 2:** Stratified pre-implementation performance at different observed Cp for individually tested specimens.

Specimens tested in a 4-specimen pool	POSITIVE CATEGORY (HIGH): Expected result individual specimen with Cp <30	PPA (95% CI)
**Pooled test result**	**Positive**	
**Positive**	36	100% (90.4–100%)
**Negative**	0	
	**POSITIVE CATEGORY (LOW): Expected result individual specimen with Cp 30–35**	
**Pooled test result**	**Positive**	
**Positive**	13	92.9% (68.5–98.7%)
**Negative**	1	
	**INDETERMINATE CATEGORY: Expected result individual specimen with Cp >35**	
**Pooled test result**	**Positive**	
**Positive**	3	60% (23.1–88.2%)
**Negative**	2	

**Fig 2:**
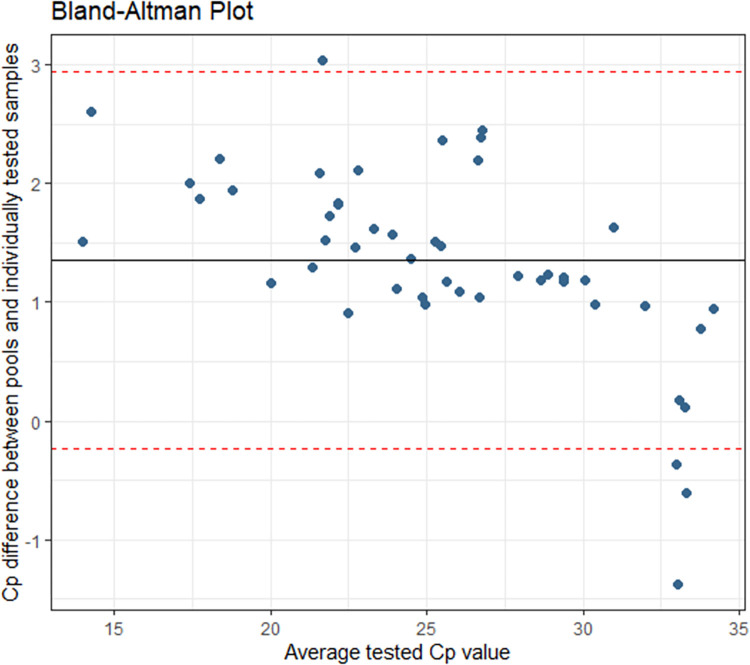
Pre-implementation: Cp agreement between individually and pooled tested specimens. The average Cp difference was 1.352 with a 95% confidence interval of -0.235 and 2.940.

This can be clearly observed in Fig 2, where the Bland-Altman agreement shows a sharp decrease in signal agreement after a Cp of 30.

**Fig 3:**
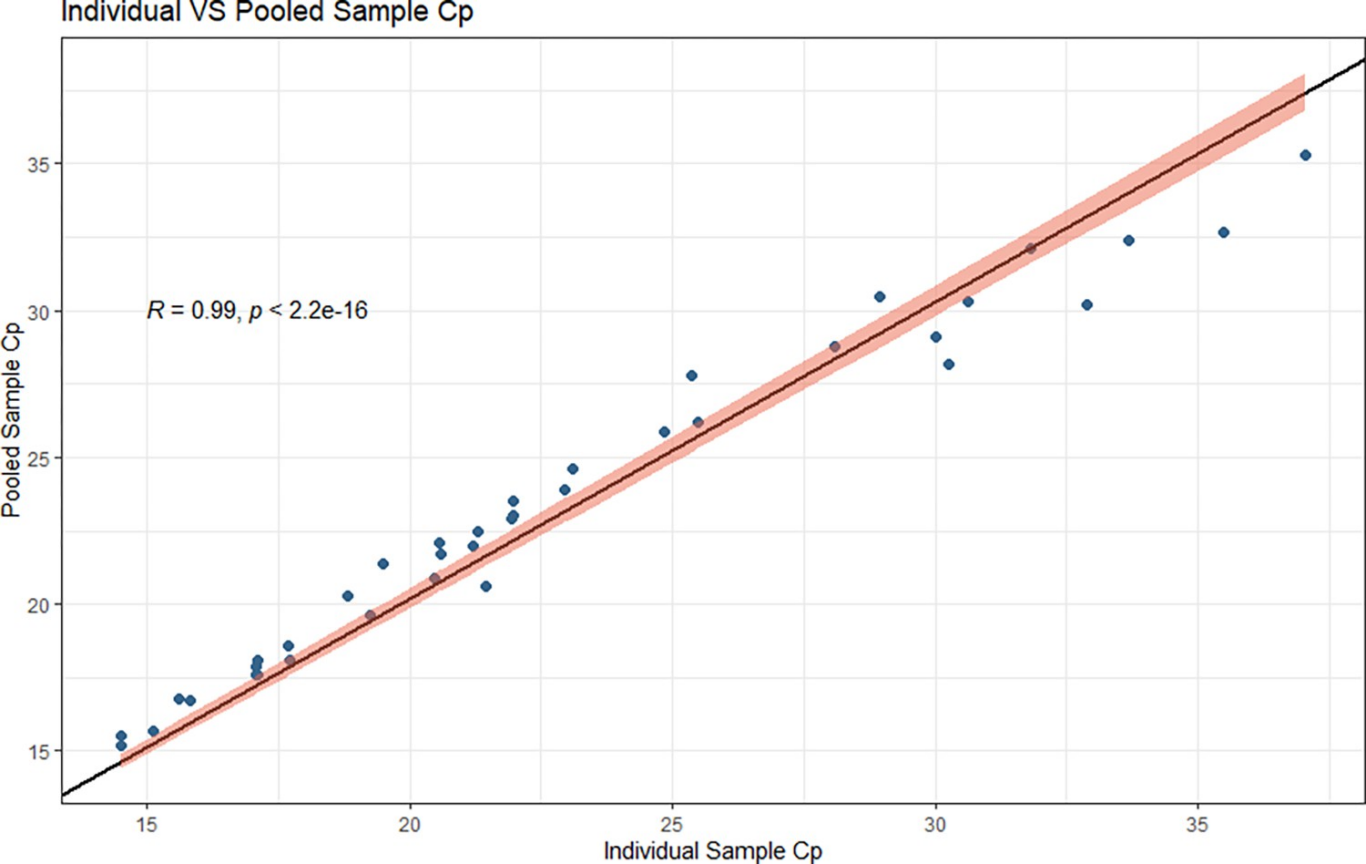
Post-implementation data plot of the individual specimen Cp and the pooled specimen Cp. Regression line is shown, y-intercept = 0, slope = 1.011, correlation of 0.9972 and a p-value of < 2.2e-16. 95% confidence interval shown in red.

**Fig 4:**
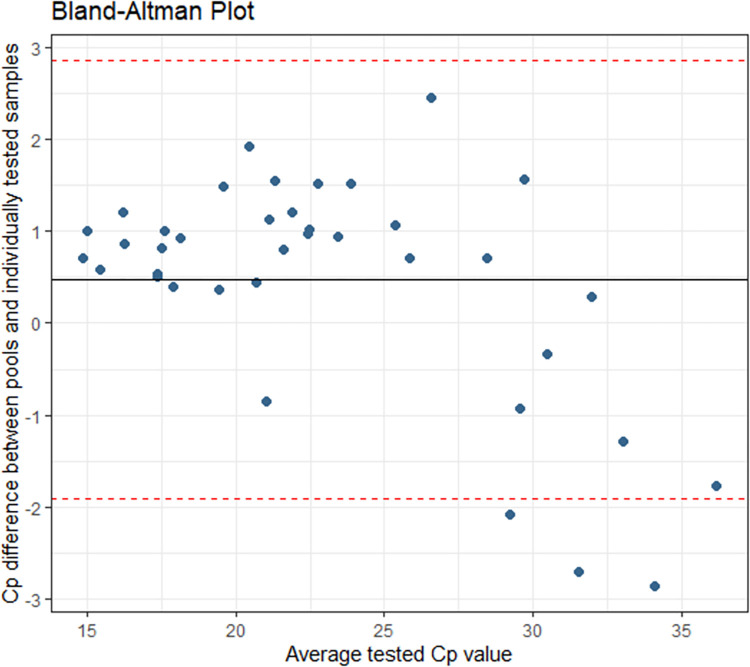
Post-implementation (1-week): Cp agreement between individually and pooled tested specimens. The average Cp difference was 0.471 with a 95% confidence interval of -1.906 and 2.847.

Performance post-implementation was in keeping with our pre-implementation data (Figs 3 and 4).

Cost-per-test impact for pooling is measured in reference to the consumable cost only and represented as a percentage reduction of cost versus the baseline cost-per-test of the individual testing stream. For example, 60% versus baseline represents 60% less consumable cost per test than if testing as individual specimens. The average cost savings for week 1 post-implementation for pooled specimens was 74% versus baseline (See Table 3 for efficiency and cost per test calculations at different positivity levels).

**Table 3:** Efficiency and cost per test at different positivity rates.

	% Positivity	% Efficiency	Cost-savings per test
**Individual testing**			Baseline
**Pooled testing**	0	400	75% reduction
	2.5	363.6	73% reduction
	5	333.3	70% reduction
	7.5	307.7	68% reduction
	10	285.7	65% reduction

A total of 10,904 specimens were resulted between September 28 and October 4, 2020 through this pooling algorithm using 2,914 tests. Total consumable cost savings were calculated to be 73.25% (Table 4).

**Table 4:** Post-implementation performance.

a) 1-week performance (September 28, 2020 –October 4, 2020)								
**Date**	**Pooled plate ID**	**Positive pools**	**Negative pools**	**Invalid pools**	**Total individual repeats**	**Total tests performed**	**Total patient specimens tested**	**Efficiency**
28-Sep	1	2	92	0	8	102	376	369%
	2	1	93	0	4	98	376	384%
	3	5	89	0	20	114	376	330%
29-Sep	4	2	92	0	8	102	376	369%
30-Sep	5	0	94	0	0	94	376	400%
	6	1	93	0	4	98	376	384%
	7	3	91	0	12	106	376	355%
1-Oct	8	3	91	0	12	106	376	355%
	9	4	90	0	16	110	376	342%
	10	2	92	0	8	102	376	369%
	11	0	94	0	0	94	376	400%
	12	2	92	0	8	102	376	369%
	13	1	93	0	4	98	376	384%
	14	0	94	0	0	94	376	400%
2-Oct	15	0	94	0	0	94	376	400%
	16	4	90	0	16	110	376	342%
	17	2	92	0	8	102	376	369%
	18	0	94	0	0	94	376	400%
3-Oct	19	1	93	0	4	98	376	384%
	20	1	93	0	4	98	376	384%
	21	1	93	0	4	98	376	384%
	22	1	93	0	4	98	376	384%
	23	3	89	2	12	106	376	347%
	24	1	93	0	4	98	376	384%
	25	0	94	0	0	94	376	400%
4-Oct	26	1	93	0	4	98	376	384%
	27	0	94	0	0	94	376	400%
	28	4	90	0	16	110	376	342%
	29	2	92	0	8	102	376	369%
**Totals**		**47**	**2,677**	**2**	**188**	**2914**	**10896**	**374%**
b) 1-week quality assurance (September 28, 2020 –October 4, 2020)								
**1-week performance**	**n**	**Rates**						
# pools	2,726							
Invalid rate	2	0.07						
Positive rate	47	1.76						
Negative rate	2,677	98.20						
False positive rate*	2	0.07						
False negative rate**	1	0.27						
c) 8-month performance (October 2020 –May 2021)								
**8-month performance**								
# pools		38,070						
# repeats		12,527						
Total tests with pooling		50,537						
Total specimens tested		152,280						
% Cost reduction through pooling		85.5%						

*Positive pools where no individual specimen is found to be positive

**Random selection for individual testing of specimens that were originally pooled (5% weekly)

Furthermore, during the 8-months that our laboratory used pooling for testing, we saw 85.5% cost-reduction and savings from our operational budget without compromising assay sensitivity and our standard of care.

## Discussion

Our validation suggests that SARS-CoV-2 in a single positive clinical specimen can be detected with a 94.5% positive percent agreement when pooled four-in-one (Table 1) and demonstrates a strong correlation between the crossing point observed from specimens tested individually and when pooled (Fig 1). Yelin and collaborators evaluated different pooling sizes and found 95% sensitivity in pooling specimens up to 32-in-one and an average increase cycle threshold (Ct) of 1.24 with every dilution by a factor of 2 [16]. Comparable studies were four-in-one specimen pools were constructed showed a correlation with individual testing of 91.4 to 100% [17–20]. Another study found that sensitivity is not impacted negatively with ten-in-one pooling with 1–2 positives with a Ct < 35. In contrast, higher Ct values resulted in >10% loss in sensitivity [10]. This observation agrees with our own during this evaluation and post-implementation, where the positive percent agreement drops to 60% for specimens with Ct > 35 (Table 2). Other studies assessing four-in-one specimen pooling have reported similar findings with a decrease of sensitivity to 77.8% [18]. Torres also evaluated the pooling size of five- and ten-in-one and compared the different Ct values for E and RdRp genes, with false negatives observed at Ct value ≥35.8 [21]. There was an expected reduction in signal translating to a 1.352 cycle delay for the pooled specimen given the 1:2 dilution effect (Fig 2). Other studies that introduced a four-in-one pooling algorithm have come to similar conclusions with an average 2 Ct loss in analytical sensitivity [22–24].

A cost analysis was performed based on the positivity rate as seen in Table 3. Even at a positivity rate of 10%, pooling was found to be cost-effective. Others have reported similar observations [11]. It is estimated that testing capacity increases by 69% with a 10% positivity rate. Pooling allowed us to increase our capacity from 3,000 to more than 6,000 tests per day with limited staff and instrumentation. Added capacity is one of the most significant advantages of pooling, especially when the population positivity rate is low during the troughs between pandemic waves and when consumable supply chains are under stress. Implementation also does not require new training or equipment; however, managing the digital information through the workflow can prove challenging without custom automated solutions. Turnaround time (TAT) is improved for negative specimens in situations of high demand versus capacity given the ability to process specimens in parallel. However, there is a compromise in TAT for the positive specimens where reflex testing is required. This may create a challenge in certain public health and clinical situations, emphasizing the importance of local risk assessment and planning prior to implementation.

The FDA recommendations include monitoring of the change in positivity to adjust the approach to pooling accordingly. While pooling is not recommended when the positivity rate is high, streaming approaches may be able to direct subpopulations with lower positivity rate to continue pooling even when the overall conditions do not warrant it. As presented here, subpopulations can be based on source setting, geography, and any other easily discernable and sortable parameter which may relate to pre-test probability. Our pooling approach showed an overall positive percent agreement of 94.5% and was dynamically targeted to populations with low pre-test probability to maximize the gain from the algorithm. The disadvantage of this approach was the increased pre-analytical sorting needed to separate the streams, and the statistical analysis needed to dynamically inform refined sorting. In our laboratory, a 5% positivity rate was used to inform our pooling approach, with subpopulations below this streamed for pooling. It was found that beyond that positivity rate, the interpretation, manual retrieval of specimens for re-testing and reporting was inefficient. Any gains still being realized in efficiency, were outweighed by the added complexity and re-work. Our individual testing algorithm performed routinely in our laboratory has a repeat rate of approximately 3.7% and an invalid rate of 1.0% (6-month average, data not shown). These values have slightly fluctuated through the different SARS-CoV-2 waves. Pooling specimens with a positivity rate of < 5% had a repeat rate of average 6.45%, almost double than the individual testing stream baseline. Other identified limitations in this study were the limited sample size used for validation and the lower analytical sensitivity at Ct > 35. These limitations were circumvented by the random selection of specimens to represent the population served in our institution following FDA guidance [12] and by testing specimens from high pre-test probability populations on the individual testing stream, respectively.

Post-implementation data shows the consumable cost savings achieved through this algorithm was 85.5% after 8 months, creating both testing and resource capacity. Pooling, as presented in this algorithm, was an effective method to be used for SARS-CoV-2 testing during the current pandemic to address resource shortages and provide quick TAT for high volumes of testing without compromising testing performance. Being able to rapidly scale up to test large volumes in the population is a critical enabler to control the spread of the disease. This method was discontinued in our center in May 2021 once supply chains and demand stabilized in our laboratory, but we continue to retain this as a viable option for the future.

The data presented here is an example of how laboratories can use innovation in testing algorithms to address seemingly uncontrollable challenges as seen during emergencies like the current pandemic such as global supply chain shortages in plastics and consumables, and demand massively exceeding available capacity. As we face further pandemic waves with different “Variants of Concern” circulating, we and other laboratories remain under pressure to maximize testing capacity and improve turnaround time. This study showcases specimen pooling as an accurate and reliable tool for the diagnosis of SARS-CoV-2 in settings experiencing testing resource constraints where the benefits of such algorithm outweigh the outlined disadvantages.

## Supporting information

S1 Fig Extraction efficiency of a larger volume of specimen, 400 μl specimen input Cp and 200 μl specimen input Cp. Regression line is shown, y-intercept = 0, slope = 0.923, correlation of 0.9768 and a p-value of < 2.2e-16. 95% confidence interval shown in red.
(TIF)

S1 Table Cp of specimens used to validate extraction efficiency of different volumes used to build S1 Fig. (DOCX)

S2 Table Overall Cp distribution of positive specimens tested individually in our laboratory between the period of September 2020 to May 2021. (DOCX)

S3 Table Cp distribution of specimens included in the pooling algorithm validation including data used to build Figs 1 and 2. (DOCX)

S4 Table Post-implementation (1-week) specimen Cps used to build Figs 3 and 4. (DOCX)
