# Supplementary figures and images for: Increasing SARS-CoV-2 testing capacity through specimen pooling: An acute care center experience
Source: PLoS One. 2023 Jun 28;18(6):e0267137. doi: 10.1371/journal.pone.0267137 (PMC10306409; doi:10.1371/journal.pone.0267137)

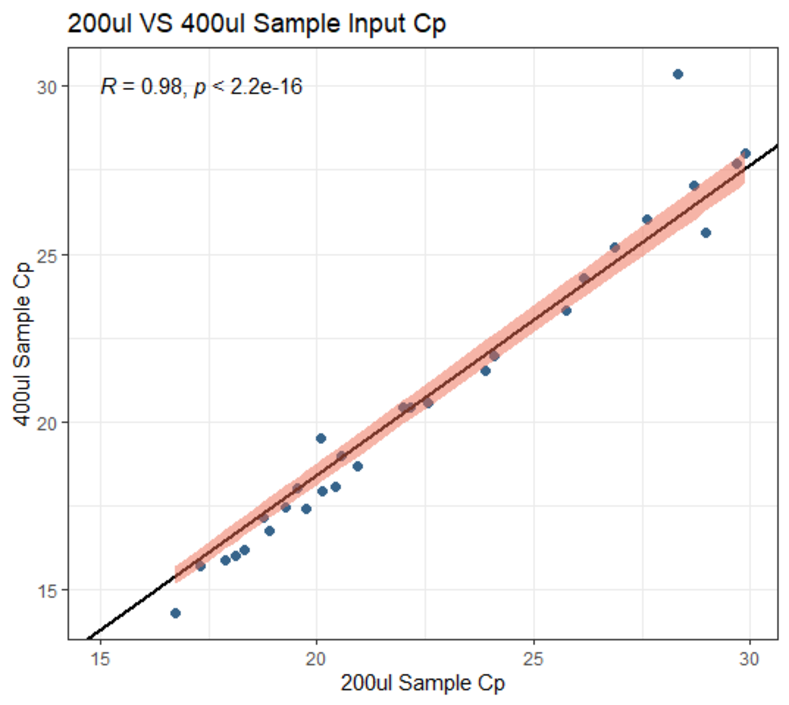

Supplement: S1 Fig — Regression line is shown, y-intercept = 0, slope = 0.923, correlation of 0.9768 and a p-value of < 2.2e-16. 95% confidence interval shown in red. (TIF) [file pone.0267137.s001.tif]
