# Supplementary material for: Increasing SARS-CoV-2 testing capacity through specimen pooling: An acute care center experience
Source: PLoS One. 2023 Jun 28;18(6):e0267137. doi: 10.1371/journal.pone.0267137 (PMC10306409; doi:10.1371/journal.pone.0267137)
Supplement: S1 Table — (DOCX) [file pone.0267137.s002.docx]

**S1 Table. Cp of specimens used to validate extraction efficiency of different volumes used to build S1 Fig.**

| **Specimen #** | **200 µl** | **400 µl** |
| --- | --- | --- |
| 1 | 17.29 | 15.7 |
| 2 | 22 | 20.42 |
| 3 | 20.55 | 19 |
| 4 | 28.98 | 25.65 |
| 5 | 24.09 | 21.96 |
| 6 | 16.73 | 14.33 |
| 7 | 18.11 | 16.03 |
| 8 | 28.33 | 30.34 |
| 9 | 20.1 | 19.51 |
| 10 | 25.76 | 23.32 |
| 11 | 18.33 | 16.19 |
| 12 | 20.43 | 18.09 |
| 13 | 19.27 | 17.48 |
| 14 | 19.74 | 17.43 |
| 15 | 17.89 | 15.89 |
| 16 | 29.89 | 28 |
| 17 | 26.15 | 24.28 |
| 18 | 22.56 | 20.55 |
| 19 | 18.77 | 17.14 |
| 20 | 18.9 | 16.76 |
| 21 | 22.14 | 20.43 |
| 22 | 20.92 | 18.68 |
| 23 | 27.61 | 26.04 |
| 24 | 26.86 | 25.2 |
| 25 | 20.11 | 17.93 |
| 26 | 29.68 | 27.71 |
| 27 | 28.68 | 27.02 |
| 28 | 19.53 | 18.04 |
| 29 | 23.89 | 21.51 |
