# Supplementary material for: Increasing SARS-CoV-2 testing capacity through specimen pooling: An acute care center experience
Source: PLoS One. 2023 Jun 28;18(6):e0267137. doi: 10.1371/journal.pone.0267137 (PMC10306409; doi:10.1371/journal.pone.0267137)
Supplement: S2 Table — (DOCX) [file pone.0267137.s003.docx]

# S2 Table. Overall Cp distribution of positive specimens tested individually in our laboratory between the period of September 2020 to May 2021.

| **<25** | **25-30** | **30-35** | **>35** | **Total samples tested Sept 2020 to May 2021** |
| --- | --- | --- | --- | --- |
| 2893 | 848 | 757 | 161 | 4,659 |
