# Supplementary material for: Increasing SARS-CoV-2 testing capacity through specimen pooling: An acute care center experience
Source: PLoS One. 2023 Jun 28;18(6):e0267137. doi: 10.1371/journal.pone.0267137 (PMC10306409; doi:10.1371/journal.pone.0267137)
Supplement: S3 Table — (DOCX) [file pone.0267137.s004.docx]

# S3 Table. Cp distribution of specimens included in the pooling algorithm validation including data used to build Fig. 1 and 2.

| **Specimen #** | **Individually tested** | **Tested in pools of 4** |
| --- | --- | --- |
| 1 | 12.89 | 15.34 |
| 2 | 13.91 | 15.54 |
| 3 | 14.23 | 15.72 |
| 4 | 15.67 | 17.54 |
| 5 | 16.31 | 17.99 |
| 6 | 16.83 | 18.52 |
| 7 | 17.65 | 17.94 |
| 8 | 18.25 | 19.14 |
| 9 | 19.14 | 20.46 |
| 10 | 20.11 | 21.51 |
| 11 | 20.24 | 21.58 |
| 12 | 20.63 | 21.82 |
| 13 | 21 | 22.51 |
| 14 | 21.27 | 22.67 |
| 15 | 21.68 | 22.91 |
| 16 | 22.57 | 24.32 |
| 17 | 22.66 | 24.89 |
| 18 | 22.77 | 24.04 |
| 19 | 23.14 | 24.31 |
| 20 | 23.42 | 24.53 |
| 21 | 23.71 | 24.9 |
| 22 | 24.05 | 25.22 |
| 23 | 24.18 | 25.73 |
| 24 | 24.3 | 25.89 |
| 25 | 24.84 | 26.21 |
| 26 | 25.5 | 26.67 |
| 27 | 25.99 | 28.61 |
| 28 | 27.22 | 27.29 |
| 29 | 28 | 29.02 |
| 30 | 28.19 | 29.61 |
| 31 | 28.49 | 32.59 |
| 32 | 28.69 | 30.31 |
| 33 | 28.93 | 30.95 |
| 34 | 29.54 | 31.7 |
| 35 | 29.59 | 30.62 |
| 36 | 29.74 | 30.76 |
| 37 | 30.44 | 33.03 |
| 38 | 30.47 | 31.32 |
| 39 | 30.56 | 31.84 |
| 40 | 30.69 | 33.91 |
| 41 | 30.79 | 32.93 |
| 42 | 31.61 | 31.18 |
| 43 | 32.05 | 33.04 |
| 44 | 32.13 | ND |
| 45 | 32.24 | 31.53 |
| 46 | 32.56 | 33.3 |
| 47 | 33.02 | 33.3 |
| 48 | 33.17 | 33.66 |
| 49 | 34.05 | 32.96 |
| 50 | 34.66 | 35.3 |
| 51 | 35.09 | 33.04 |
| 52 | 35.8 | 34.68 |
| 53 | 36.58 | ND |
| 54 | 37.21 | 37.65 |
| 55 | 39 | ND |

# ND: not detected
