# Supplementary material for: Increasing SARS-CoV-2 testing capacity through specimen pooling: An acute care center experience
Source: PLoS One. 2023 Jun 28;18(6):e0267137. doi: 10.1371/journal.pone.0267137 (PMC10306409; doi:10.1371/journal.pone.0267137)
Supplement: S4 Table — (DOCX) [file pone.0267137.s005.docx]

**S4 Table. Post-implementation (1-week) specimen Cps used to build Fig. 3 and 4.**

| **Specimen #** | **Individually tested** | **Tested in pools of 4** |
| --- | --- | --- |
| 1 | 37.06 | 35.29 |
| 2 | 17.08 | 17.9 |
| 3 | 21.93 | 22.9 |
| 4 | 19.48 | 21.4 |
| 5 | 19.24 | 19.6 |
| 6 | 22.96 | 23.9 |
| 7 | 17.06 | 17.6 |
| 8 | 30.64 | 30.3 |
| 9 | 35.5 | 32.65 |
| 10 | 30.27 | 28.2 |
| 11 | 32.9 | 30.2 |
| 12 | 17.68 | 18.6 |
| 13 | 17.7 | 18.1 |
| 14 | 14.5 | 15.2 |
| 15 | 25.5 | 26.2 |
| 16 | 21.3 | 22.5 |
| 17 | 14.5 | 15.5 |
| 18 | 15.6 | 16.8 |
| 19 | 21.2 | 22 |
| 20 | 17.1 | 17.6 |
| 21 | 28.1 | 28.8 |
| 22 | 23.09 | 24.6 |
| 23 | 24.84 | 25.9 |
| 24 | 18.82 | 20.3 |
| 25 | 20.58 | 21.7 |
| 26 | 28.93 | 30.5 |
| 27 | 17.09 | 18.1 |
| 28 | 15.83 | 16.7 |
| 29 | 15.11 | 15.7 |
| 30 | 21.98 | 23 |
| 31 | 21.45 | 20.6 |
| 32 | 30.02 | 29.1 |
| 33 | 21.98 | 23 |
| 34 | 20.46 | 20.9 |
| 35 | 20.56 | 22.1 |
| 36 | 25.35 | 27.8 |
| 37 | 31.82 | 32.1 |
| 38 | 33.69 | 32.4 |
| 39 | 21.98 | 23.5 |
